# Transitioning to DNA genomes in an RNA world

**DOI:** 10.7554/eLife.32330

**Published:** 2017-11-01

**Authors:** Razvan Cojocaru, Peter J Unrau

**Affiliations:** Department of Molecular Biology and BiochemistrySimon Fraser UniversityBurnabyCanada

**Keywords:** ribozyme, reverse transcriptase, polymerase, origins of life, None

## Abstract

The unexpected ability of an RNA polymerase ribozyme to copy RNA into DNA has ramifications for understanding how DNA genomes evolved.

**Related research article** Samanta B, Joyce GF. 2017. A reverse transcriptase ribozyme. *eLife* **6**:e31153. doi: 10.7554/eLife.31153

For as long as history has been recorded, humanity has tried to answer the ancient question of our origins. The ‘central dogma’ of molecular biology, first stated by Francis Crick in 1958, represented a major step forward in our efforts to answer this question (Figure 1A; Crick, 1958). In this model, the genetic information stored in DNA is transcribed to produce RNA, which is then translated by the ribosome to produce chains of amino acids. These chains fold to make the proteins that are responsible for almost everything that happens in cells.

**Figure 1.**
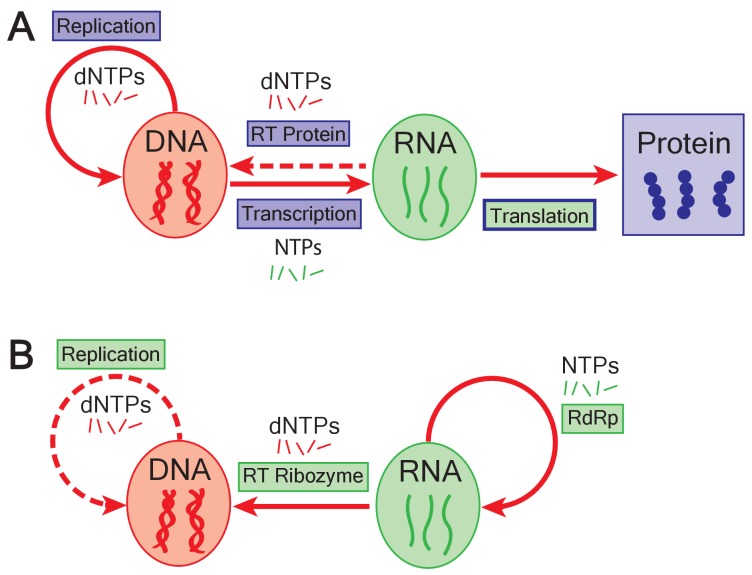
The emergence of DNA genomes in the RNA world. (**A**) In the central dogma of molecular biology, information flows from DNA (red oval) to RNA (green oval) to protein (blue box). DNA is formed of building blocks called deoxynucleoside triphosphates (dNTPs) and can be replicated (solid looping red arrow); RNA is formed of nucleoside triphosphates (NTPs). Enzymes called reverse transcriptases (RT) enable complementary DNA to be made from the building blocks of RNA (dashed arrow). Blue rectangles represent processes catalyzed by proteins; green rectangles show processes catalyzed by RNA; translation is mediated by an RNA catalyst (green inner rectangle) that has proteins that modulate its activity (blue outline). (**B**) In the RNA world, ribozymes (RdRp) replicate RNA genomes (solid looping red arrow). Based on the work of Joyce and Samanta, if dNTPs were present in the RNA world, reverse transcriptase ribozymes could have constructed DNA genomes using RNA genomes as a template (straight red arrow). Ribozymes could also have potentially replicated DNA genomes (dashed red arrow).

The flow of information from DNA to RNA to protein is thought to have evolved out of a simpler evolutionary period when genetic information was stored and transmitted solely by RNA molecules. This theory, known as the ‘RNA world hypothesis’, posits that an RNA enzyme or ‘ribozyme’ capable of copying RNA molecules existed early in evolution, and that protein synthesis by the ribosome (which is also an RNA enzyme) evolved out of this system (Figure 1B; Gilbert, 1986; Atkins et al., 2011). The theory, however, is largely silent on how DNA genomes evolved.

In modern metabolism, protein-based enzymes called reverse transcriptases can copy RNA to produce molecules of complementary DNA. Other enzymes can promote the production of DNA nucleotides (the building blocks of DNA molecules) from RNA nucleotides via challenging chemical reactions. So how did the first DNA genomes come to be? There are two possibilities within the framework of the RNA world. In the first, protein enzymes evolved before DNA genomes. In the second, the RNA world contained RNA polymerase ribozymes that were able to produce single-stranded complementary DNA and then convert it into stable double-stranded DNA genomes.

A number of laboratories around the world are trying to build ribozymes that can sustain RNA replication (Wang et al., 2011; Attwater et al., 2013). Recently, David Horning and Gerald Joyce artificially evolved a ribozyme that is capable of copying complex RNAs and amplifying short RNA templates (Horning and Joyce, 2016). Now, in eLife, Joyce and Biswajit Samanta at the Salk Institute demonstrate that this ribozyme is also a reverse transcriptase (Samanta and Joyce, 2017). Feeding DNA nucleotides to this ribozyme enabled it to copy short segments of RNA templates into complementary DNA. This suggests that if an RNA world contained DNA nucleotides, DNA genomes could have been assembled and then presumably replicated by ribozymes.

Whether DNA genomes existed very early in evolution fundamentally rests on whether DNA nucleotides were available in the RNA world. There are plausible routes by which RNA and DNA nucleotides could have been synthesized before life emerged, meaning that they are likely to have been available at the dawn of an RNA world (Ritson and Sutherland, 2014; Becker et al., 2016; Kim and Benner, 2017). Likewise, artificially selected ribozymes have been used to synthesize the two types of bases found in RNA nucleotides from simpler precursors, suggesting RNA nucleotides could have been built by early RNA systems (Martin et al., 2015). If DNA precursors were also available early in evolution, then the synthesis of DNA nucleotides by an RNA system appears likely. While this area is currently underexplored experimentally, there appears to be no fundamental reason why DNA nucleotides could not have been abundant quite early in evolution.

Demonstrating that DNA polymerase ribozymes are able to rapidly use such DNA nucleotides would represent a major step forward for the early DNA genome model. While the field of artificial RNA polymerase ribozymes has made rapid strides, their ability to add multiple nucleotides rapidly is still very limited. Current ribozymes are significantly longer and more complex than the sequences that they are able to copy, but to make self-evolving systems, ribozymes need to be able to copy sequences that are longer and more complex than themselves. It will therefore be exciting to see if the techniques that have created such RNA polymerases are also able to evolve DNA polymerase ribozymes that have the potential to make self-replicating systems using DNA and not RNA as a source of genetic material. Such a system would bring us closer to understanding the transition from an RNA world to a type of life that respects the rules of the central dogma of modern biology.
